# Antioxidant and Hypoglycemic Activity of Sequentially Extracted Fractions from Pingguoli Pear Fermentation Broth and Identification of Bioactive Compounds

**DOI:** 10.3390/molecules27186077

**Published:** 2022-09-17

**Authors:** Jing Dai, Yu Hu, Qi Si, Yifei Gu, Zhuqian Xiao, Qin Ge, Ruyi Sha

**Affiliations:** 1 School of Biological and Chemical Engineering, Zhejiang University of Science and Technology, Hangzhou 310023, China; 2 Zhejiang Provincial Key Laboratory for Chemical and Biological Processing Technology of Farm Products, Zhejiang Provincial Collaborative Innovation Center of Agricultural Biological Resources Biochemical Manufacturing, Hangzhou 310023, China

**Keywords:** Pingguoli pear fermentation broth, antioxidant, hypoglycemic activity, sequential extraction, LC–MS analysis

## Abstract

Pear fruits have been reported to contain abundant bioactive compounds and exhibit antidiabetic activity. In this study, Pingguoli pear (*Pyrus pyrifolia* cv.‘Pingguoli’) fermentation broth was sequentially extracted by five solvents with increasing polarity (petroleum ether, chloroform, ethyl acetate, n-butanol, and water) to evaluate its antioxidant and hypothermic activities, and then the main compounds of the fraction with the highest activity were assessed, which might be responsible for such activities. The results showed that the ethyl acetate fraction (EAF) exhibited the highest antioxidant activity according to DPPH (IC_50_ = 0.238 mg/mL), ABTS (IC_50_ = 0.293 mg/mL), and FRAP (IC_50_ = 0.193 mg/mL) assays. The in vitro hypoglycemic activity assay showed that EAF exhibited the strongest inhibitory effect, with IC_50_ values of 0.34 and 0.95 mg/mL for α-amylase and α-glucosidase, respectively. The glucose consumption in HepG2 cells treated with EAF was significantly increased to 252%, compare with control group. Liquid chromatography–mass spectrometry analysis implied that the main compounds, 3′-C-glucosylisoliquiritigenin, robustside D, caffeic acid, and chlorogenic acid may be potential candidates for the antioxidant and hypoglycemic activities of the EAF. This study suggested that EAF of Pingguoli pear fermentation broth could be utilized for development of potential functional food and antidiabetic agents.

## 1. Introduction

Diabetes is a metabolic disease with multiple etiologies, mainly manifested by an increase in blood glucose levels and disordered glucose metabolism, followed by disorders of protein and fat metabolism, leading to cardiovascular and cerebrovascular diseases, renal failure, retinopathy, etc. [1]. The chemical drugs commonly used to treat diabetes have been reported to show certain toxic and side effects on the human body, such as inducing liver and kidney damage and intestinal discomfort [2]. In recent years, anti-diabetic functional foods and drugs that contain natural active ingredients with little or no side effects have attracted widespread attention. These natural active ingredients show mild and durable effects on blood glucose and lipids reduction, and glucose tolerance improvement [3]. Several mechanisms underlying the antidiabetic activities of some plants and dietary compounds have been reported, including the stimulation of insulin secretion from pancreatic β-cells, increasing insulin sensitivity, the stimulation of AMP-activated protein kinase activity, the regulation of glucose metabolism and lipid metabolism, and the inhibition of α-amylase and α-glucosidase [4,5,6].

Fresh pear fruits have been reported to contain abundant vitamins, minerals, polyphenols, flavonoids, anthocyanins, and triterpenes, and thus exhibit a broad spectrum of pharmacological activities, such as antioxidant, hypoglycemic, anti-inflammatory, antiulcer, anti-cancer, cardiovascular, and cerebrovascular effects [7,8]. α-amylase and α-glucosidase are the key enzymes that affect the digestion and absorption of carbohydrates in the diet. Inhibiting their activities can delay the degradation of carbohydrates and the absorption of glucose by the human body, thus inhibiting the rapid postprandial rise in blood glucose [4]. Barbosa et al. found that aqueous and ethanolic extracts of the peel and pulp from different varieties of pears showed varying degrees of antioxidant and α-amylase- and α-glucosidase-inhibitory activities, which were positively correlated with their total phenol contents. However, the strong inhibitory activity of an aqueous extract of pear pulp on α-amylase was not related to its total phenol content, suggesting that there may be other active hypoglycemic ingredients [9]. Xiang Ting et al. studied the hypoglycemic effect of Tangli pear and found that, in a streptozotocin-induced diabetes mouse model, Tangli pear reduced the fasting blood glucose and improved the glucose tolerance of diabetic mice [10]. On the other hand, although the pear peel is rich in various nutrients and active ingredients, its poor sensory flavor reduces its typical consumption. Therefore, pear peel is usually treated as waste product during processing or eating, resulting in a large loss of nutrients. Wang et al. analyzed the compounds of the eight most widely distributed pear varieties in China, and found that the pear peel contained higher levels of phenolic acids, flavonoids, and triterpenes than the pear pulp, and their antioxidant activity was positively correlated with the contents of phenolic acid and flavonoids. Furthermore, the pear peel showed higher inhibitory activity for α-glucosidase than pulp, which was speculated to be related to the high amounts of polyphenols from the peel, including chlorogenic acid, vanillic acid, ferulic acid, and rutin. Their further study on the streptomycin-induced type I diabetes mouse model showed that pear peel could better reduce the fasting blood glucose level, alleviate oxidative stress and blood lipid accumulation, and improve glucose tolerance [11].

Microbial fermentation technology can not only decompose the non-digestible macromolecular substances in food into digestible small molecules, but also produce a series of active metabolites, which has the advantages of improving food flavor quality and enhancing nutritional value [12]. Ankolekar et al. used *Lactobacillus acidophilus* to ferment four commonly consumed cultivars of pears (Anjou, Red Anjou, Bosc, and Comice) for 72 h. The results showed that the inhibitory activity of the fermented pear juice against α-glucosidase increased in a dose-dependent manner with the prolongation of the fermentation time [13]. Therefore, the microbial fermentation of pear fruits can not only improve the overall utilization of the pear peel and pulp, but might also enhance their antidiabetic effects.

The Pingguoli pear (*Pyrus pyrifolia* cv. ‘Pingguoli’) is a characteristic fruit in Yanbian Prefecture and is one of the main pear varieties in northern China. It is a red skin cultivar with an apple shape and has excellent storage life. It is rich in amino acids, trace elements, vitamins, polyphenols, flavonoids, and other nutrients [14]. Its taste is crisp, juicy and sweet–sour. At present, the conventional products of Pingguoli pear are mainly fresh and dried fruits. Thus, it is necessary to develop an effective processing method to exploit high value-added products of Pingguoli pear. To the best of our knowledge, previous research has mainly focused on the antifungal and antioxidant activity of Pingguoli pears [14,15]; the hypoglycemic activity of the Pingguoli pear has not been reported yet. In our previous study, we conducted a fermentation study on Pingguoli pears and found that the contents of phenols, flavonoids, and proanthocyanidins in Pingguoli pear fermentation broth, as well as the antioxidant and α-glucosidase inhibitory activity of the fermentation broth, increased significantly with the prolongation of the fermentation time [16].

Therefore, to identify the antioxidant and hypoglycemic compounds in Pingguoli pear fermentation broth and to gain insight into the mechanism underlying the hypoglycemic effect of active compounds, in this study, Pingguoli pear fermentation broth was sequentially extracted with five polar solvents (petroleum ether, chloroform, ethyl acetate, n-butanol, and water) to obtain five extracted fractions. The antioxidant activity of each extracted fraction was evaluated by 2,2-diphenyl-1-picrylhydrazyl (DPPH) and 2,2′-amino-di(2-ethyl-benzothiazoline sulphonic acid-6)ammonium salt (ABTS) radical scavenging assay, and ferric reducing antioxidant power (FRAP) assay. The hypoglycemic activity of each extracted fraction was evaluated by assaying the inhibitory activities against α-amylase and α-glucosidase, and the glucose consumption of a hepatoma cell line (HepG2) and insulin-resistant (IR) HepG2 cells. Finally, the chemical composition of the fraction with the highest activity was identified by liquid chromatography–mass spectrometry analysis (LC–MS/MS) to characterize the potential compounds responsible for the antioxidant and hypoglycemic effects.

## 2. Results and Discussion

### 2.1. Content of Each Extracted Fraction

Pingguoli pear fermentation broth was sequentially extracted using petroleum ether, chloroform, ethyl acetate, n-butanol, and water to obtain five extracted fractions: the petroleum ether fraction (PEF), chloroform fraction (CF), ethyl acetate fraction (EAF), n-butanol fraction (NF), and water fraction (WF). The sequential extraction method ensured the extraction of active compounds from the fermentation broth according to their polarity. The total content of various compounds extracted by each solvent is shown in Table 1. The WF showed the highest content of 573.23 mg per mL Pingguoli pear fermentation broth, which means that 573.23 mg of dry extract was obtained from 1 mL of Pingguoli pear fermentation broth by water extraction. The fact that the highest content was obtained in the WF extract indicated abundant polar compounds in the Pingguoli pear fermentation broth, according to the principle of similar solubility.

### 2.2. In Vitro Antioxidant Activities of Each Extracted Fraction

**Figure 1:**
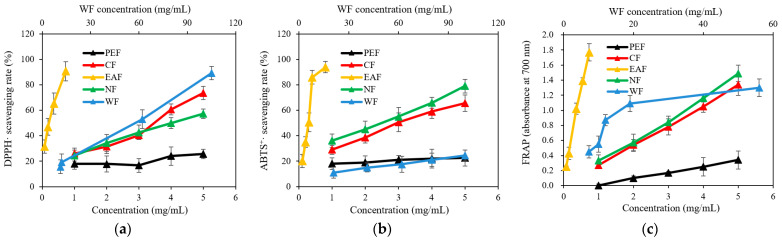
Antioxidant activity of the five extracted fractions from Pingguoli pear fermentation broth with different concentrations, including DPPH free radical scavenging rate (**a**), ABTS free radical scavenging rate (**b**) and FRAP (**c**). Values are showed as the mean ± SD (*n* = 3).

The DPPH and ABTS free radical scavenging capacities of each fraction are presented in Figure 1a,b. All the extracted fractions displayed dose-dependent antioxidant activity. Among them, the EAF showed a significant free radical scavenging rate of more than 90% at 1.0 mg/mL, while the WF showed the lowest rate of 89% at 105.0 mg/mL and 25% at 100.0 mg/mL for DPPH and ABTS, respectively. In order to compare the free radical scavenging activity, the half inhibitory concentration (IC_50_)—the concentration at which the free radical scavenging rate was 50%—was employed to evaluate the antioxidant activities of the different extracted fractions. As shown in Table 2, although the activity of all the extracted fractions was weaker than that of the standard antioxidant ascorbic acid, the EAF showed the strongest DPPH free radical scavenging activity (IC_50_ = 0.238 ± 0.052 mg/mL). The extracted fractions in descending order of scavenging activity according to the IC_50_ were EAF > CF > NF > PEF > WF. The scavenging activity of each extract for ABTS free radicals was the same as that for DPPH, and the EAF showed high free radical scavenging activity (IC_50_ = 0.293 ± 0.032 mg/mL). The extracted fractions in descending order of scavenging activity according to the IC_50_ were EAF > NF > CF > PEF > WF. As shown in Figure 1c and Table 2, the extracted fractions in descending order of reducing power (FRAP) were EAF > NF > CF > PEF > WF, which was consistent with the results for the free radical scavenging ability. The EAF also showed the highest activity, with IC_50_ = 0.193 ± 0.022 mg/mL. According to previous literature, ethyl acetate favors the extraction of many phenolic compounds, such as flavonoids, which have been reported to exhibit strong antioxidant activity [17,18]. Based on the results of the antioxidant activity analysis, the EAF showed better activity, possibly due to the higher contents of phenolics and flavonoids, which strongly contribute to electron donations that terminate radical chain reactions by quenching free radicals and reactive oxygen species (ROS) into more stable products [19].

Free-radical species such as reactive oxygen species play an important role in the pathogenesis of diabetes. Chronic exposure to hyperglycemia can induce the generation of reactive radicals due to glucose oxidation, glucose toxicity, and oxidative phosphorylation, and thus stimulate the dysfunction and destruction of β-cells, leading to the impairment of insulin function and diabetes [20]. Chronic oxidative stress can also impair the endogenous antioxidant defense system in diabetic patients and is involved in some diabetic complications, such as diabetic nephropathy, diabetic retinopathy, and diabetic neuropathy [21]. Therefore, it is speculated that the extracted fraction with the strongest antioxidant activity might possess potential hypoglycemic activity.

### 2.3. Hypoglycemic Activities of Each Extracted Fraction

#### 2.3.1. α-Amylase and α-Glucosidase Inhibitory Activities

**Figure 2:**
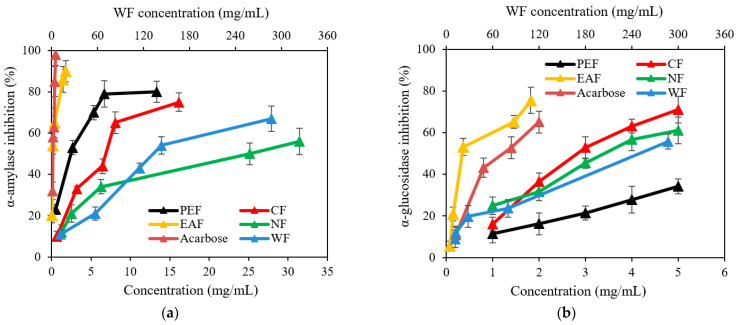
α-Amylase inhibitory effects (**a**) and α-glucosidase inhibitory effects (**b**) of each extracted fractions from Pingguoli pear fermentation broth with different concentrations. Values are mean ± SD (*n* = 3).

As shown in Figure 2, all the fractions showed inhibitory activities against α-amylase and α-glucosidase in a concentration-dependent manner. According to the IC_50_ values shown in Table 3, the order of the inhibition of α-amylase activity was EAF > PEF > CF > NF > WF. The order of the inhibitory ability for α-glucosidase activity was EAF > CF > NF > PEF > WF. This indicated that the EAF presented strong inhibitory activities against both carbohydrate hydrolases, with IC_50_ values of 0.34 ± 0.04 and 0.95 ± 0.19 mg/mL for α-amylase and α-glucosidase, respectively, comparable with acarbose (reference standard).

#### 2.3.2. Glucose Consumption in HepG2 Cells and IR-HepG2 Cells

A prolonged hyperglycemic condition leads to excessive secretion of insulin by pancreatic β-cells for prompting the body’s cells to take up the glucose in the plasma and reduce the blood glucose concentration. Chronic exposure to excess insulin may decrease the insulin sensitivity of various tissues and cells, and induce IR [22]. Type II diabetes accounts for about 90–95% of diabetes cases, and IR is a key feature of type II diabetes [23]. In this study, we employed HepG2 cells and IR-HepG2 cells to investigate the regulation of cell glucose consumption by the five extracted fractions.

**Figure 3:**
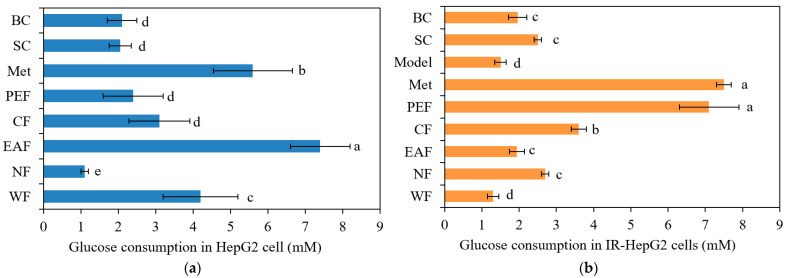
Effect of different extracted fractions from Pingguoli pear fermentation broth on glucose consumption in HepG2 cells (**a**) and IR-HepG2 cells (**b**). BC represents blank control, cells treated with medium; SC represents solvent control, cells treated with medium contain 0.5% ethanol. Values are mean ± SD (*n* = 6). Columns with different lowercase letters are significantly different (*p <* 0.05).

Firstly, the effect of the extracted fractions on HepG2 cell viability was assessed. As shown in Table 4, at their maximum nontoxic concentration, there were no significant differences in the cell viability among blank control (BC), solvent control (SC), positive control (metformin, Met), IR model, and five extracted fractions. For the glucose consumption experiments, as shown in Figure 3, the different extracted fractions showed different effects on glucose consumption in cells at their maximum nontoxic concentration. Compared with that in the control group, the glucose consumption capacity of cells treated with Met and the EAF was significantly improved. The glucose consumption of HepG2 cells with the EAF was the highest at 7.581 mM, even higher than that in the Met group (positive control, 5.819 mM), suggesting that the EAF has the potential to regulate blood glucose and prevent diabetes. On the other hand, as shown in Figure 3b, the glucose consumption of the IR model was lower than that in the control group, indicating that the model of insulin resistance induced by high glucose and high fat was successfully established. Compared with that in the IR model group, the glucose consumption of IR cells treated with Met or the PEF was significantly improved, at 7.647 and 7.093 mM, respectively. However, there was no significant improvement in glucose consumption in the EAF group. It is suggested that the PEF can promote glucose consumption in IR cells and reduce the adverse effects of IR through pathways other than antioxidant pathways.

### 2.4. LC–MS Analysis of EAF

**Figure 4:**
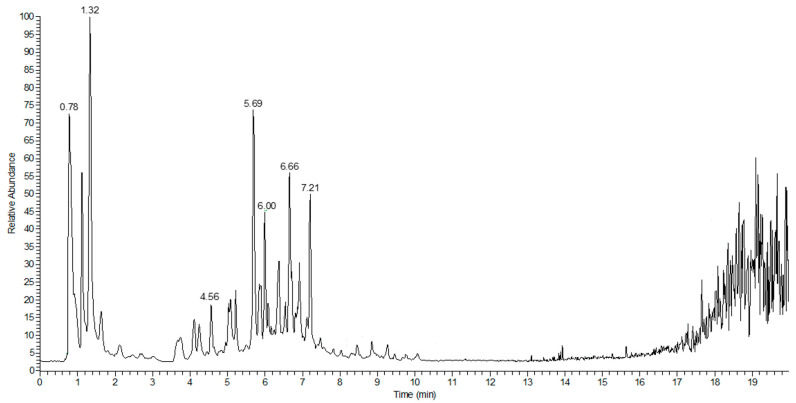
Total ion current chromatogram (TIC) of EAF from Pingguoli pear fermentation broth at negative ion mode.

**Figure 5:**
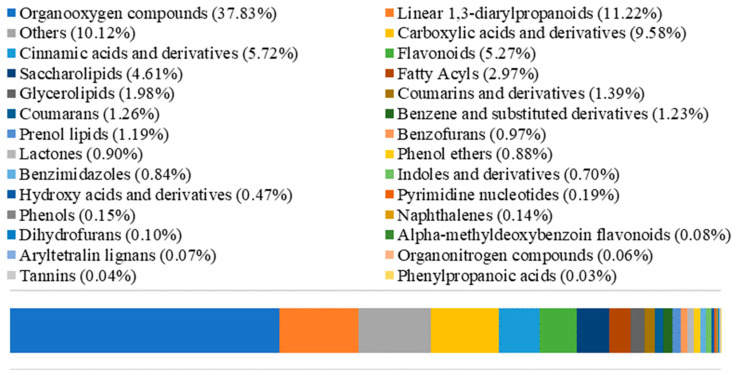
Different classifications of compounds and their relative contents found in EAF from Pingguoli pear fermentation broth. The different colors represented different classifications.

**Figure 6:**
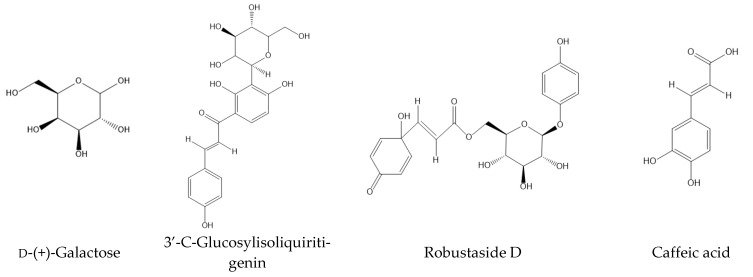

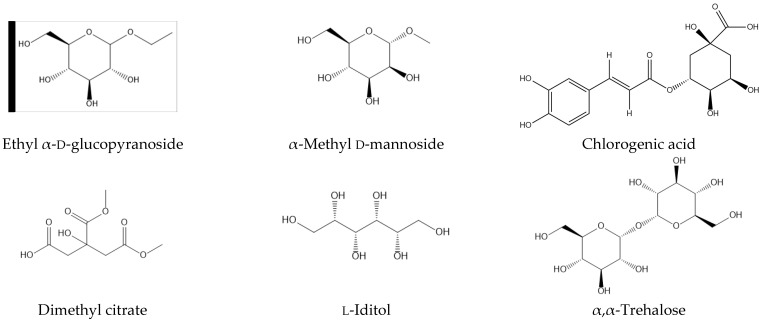
The chemical structures of ten main compounds identified in EAF from Pingguoli pear fermentation broth.

According to the results above, the EAF, which showed the highest antioxidant and hypoglycemic activities, was subjected to compound analysis by LC–MS detection. The negative ion mode was adopted due to the low pH of the EAF. The total ion flow chromatogram is shown in Figure 4. In negative ion mode, 133 compounds were identified and classified into 28 groups. The classifications and their relative contents are shown in Figure 5. The predominant compounds were organooxygen compounds (37.83%), linear 1,3-diarylpropanoids (11.22%), and carboxylic acids and derivatives (9.58%). After characteristic peak extraction and database identification, ten main compounds of the EAF, as well as their formula, category, molecular weight, retention times, and relative concentration were as listed in Table 5, and the corresponding molecular structures are shown in Figure 6. The four overlapped peaks for l-Iditol (RT = 0.763), d-(+)-Galactose (RT = 0.781), α,α-Trehalose (RT = 0.796), and α-Methyl d-mannoside (RT = 0.843) were separately quantified by dividing the area with a perpendicular to the baseline from the minimum point between the two adjacent peaks. Then, the substance identification was conducted using the database search approach according to secondary mass spectrometry information. Many of the identified compounds show valuable biological activities. 3′-C-glucosylisoliquiritigenin is a flavonoid belonging to the chalcone group that has been reported to be able to scavenge free radicals by H-atom transfer [24]. Robustside D is a phenolic glycoside that was first isolated from the natural plant *Grevillea robusta* in 2000 by Amany et al., and it shows antimalarial activity [25]. Caffeic acid is a natural phenolic compound derived from hydroxycinnamic acid, and is known to have strong antioxidant activity [26]. Caffeic acid was also reported to inhibit α-amylase and α-glucosidase activities, and improve the blood glucose of diabetic rats via increasing the expression of hepatic GK to increase the utilization of the blood glucose for glycogen storage and reducing the expression of hepatic PEPCK, G6Pase, and GLUT2 to decrease hepatic glucose production and glucose output [27,28]. Ethyl α-d-glucopyranoside belongs to the family of glycoside compounds, and it has a moisturizing effect and improves the roughness of skin [29]. Chlorogenic acid, an ester of caffeic acid with quinic acid, is a type of phenolic acid and was reported to show antioxidant and hypoglycemic effects by activating Akt pathway to improve glucose metabolism [30,31,32]. Chen et al., has reported that the synergistic effect of chlorogenic acid and caffeic acid could improve the insulin sensitivity and glucose metabolism of HepG2 cells via upregulating the protein expression of IRS-1, Akt, PI3K, and GLUT-4 [33]. Dimethyl citrate was reported to inhibit the inflammation of RAW264.7 cells [34]. Therefore, 3′-C-glucosylisoliquiritigenin, robustside D, caffeic acid, and chlorogenic acid were speculated to be responsible for the functional activities of the EAF. Furthermore, our above results indicated that these bioactive compounds might exert the antidiabetic effect through antioxidation, inhibition of α-amylase and α-glucosidase activities, or enhancing glucose consumption capacity of tissue cells.

## 3. Materials and Methods

### 3.1. Materials and Reagents

Pingguoli pears were provided by the Pingguoli pear planting base in Yanji City, Jilin Province, China. 2,2-diphenyl-1-picrylhydrazyl (DPPH), 2,2-diazo-bis-(3-ethylbenzothiazoline-6-sulfonic acid) (ABTS), and 4-Nitrophenyl-α-d-glucopyranoside (PNPG) were purchased from Sigma-Aldrich Co., Ltd. (Shanghai, China). The α-amylase kit, α-glucosidase kit, and glucose assay kit were purchased from the Nanjing Jiancheng Bioengineering Institute (Wuhan, China). HepG2 cells were purchased from Procell Life Science & Technology Co., Ltd. (Wuhan, China). Phenol red-free DMEM was purchased from Zhejiang Cienry Biotechnology Co., Ltd. (Hangzhou, China). Methanol (LC–MS grade) and acetonitrile (LC–MS grade) were purchased from Merck KGaA (Darmstadt, Germany). Formic acid (LC–MS grade) was purchased from Xiya Chemical Technology Co., Ltd. (Linyi, China). The other reagents were obtained locally and were of analytical grade.

### 3.2. Preparation of Extracted Fractions

The fresh Pingguoli pears with peels were cleaned and cut into small pieces, and then mixed with granulated sugar in a fermentation tank according to our previous research [16]. After 50 d, the Pingguoli pear fermentation broth was centrifuged at 4000 rpm for 15 min, and supernatant was successively extracted with petroleum ether, chloroform, ethyl acetate, n-butanol, and water (from small to large polarity) in a 1:1 volume at ambient temperature, and each extraction was repeated two to three times before the extracts were combined. The combined extracts were concentrated to dryness using a rotary vacuum evaporator (Eyela N-1100V-W, Tokyo Rikakikai Co. Ltd., Tokyo, Japan) at 50–75 °C or a vacuum freeze dryer (YTLG-10A, Shanghai Yetuo Technology Co., Ltd., Shanghai, China). Then, the dried extracts were weighed and dissolved in absolute ethanol or pure water to obtain the desired concentrations for subsequent use.

### 3.3. In Vitro Antioxidant Activity Assay

A DPPH free radical scavenging assay was performed using the method of Pinteus et al., with a few modifications [35]. The 2 mL sample (0.073–105 mg/mL) was mixed with 4 mL of DPPH-methanol solution (0.1 mM) and 450 µL of Tris-HCl buffer (50 mM, pH 7.4), and incubated in a water bath at 25 °C for 30 min. Deionized water and ascorbic acid were used as the control and standard antioxidant, respectively. The absorbance of the reaction mixture was recorded at 517 nm using a multi-mode microplate reader (SpectraMax^®^ iD3, Molecular Devices, San Jose, CA, USA). The scavenging effect of each extract was calculated as follows: DPPH radical-scavenging activity (%) = [(OD_control_ − OD_sample_)/OD_control_] × 100. The IC_50_ value (mg/mL), which was the concentration required to inhibit 50% of the initial DPPH free radicals, was calculated from the graph of the inhibition curve.

An ABTS free radical scavenging assay was performed using the method of Arnao et al. with a few modifications [36]. ABTS was dissolved in phosphate buffer solution (pH 7.4) and potassium persulfate to prepare the ABTS^+^ solution (2.455 mM), and was kept for 12 h at room temperature in the dark. Before use, the solution was diluted with phosphate buffer solution to an absorbance of 0.70 (±0.02) at 734 nm. A 100 µL volume of sample (0.1–100 mg/mL) was mixed with 200 µL of phosphate buffer solution and 5 mL of ABTS^+^ solution. Then, the mixed solution was incubated for 1 h at 30 °C. Deionized water and ascorbic acid were used as the control and standard antioxidant, respectively. The absorbance of the solution was recorded at 734 nm using a multi-mode microplate reader. The scavenging effect of each extract was calculated as follows: ABTS free-radical-scavenging activity (%) = [(OD_control_ − OD_sample_)/OD_control_] × 100. The IC_50_ value (mg/mL), which was the concentration required to inhibit 50% of the initial ABTS free radicals, was calculated from the graph of the inhibition curve.

FRAP was determined by the method of Tohidi et al. with a few modifications [37]. A 2.5 mL sample of phosphate buffer solution (0.073–56 mg/mL) was mixed with 2.5 mL of ferricyanide of potassium solution (10 g/L) and incubated at 50 °C for 30 min. Then, 2.5 mL of trichloroacetic acid solution (100 g/L) was added to the mixture, and it was left to stand for 10 min. The mixture was centrifuged at 3000 rpm for 20 min, before 2.5 mL of supernatant was mixed with 2.5 mL of deionized water and 0.5 mL of ferric chloride solution (1 g/L). The absorbance of the reaction mixture was measured at 700 nm using a multi-mode microplate reader. Deionized water and ascorbic acid were used as the control and standard antioxidant, respectively. An increase in the absorbance value indicated a higher reducing power. The IC_50_ was the sample extract concentration that provided an absorbance of 0.5 at 700 nm.

### 3.4. Determination of Hypoglycemic Activity

#### 3.4.1. Inhibitory Activities of α-Amylase and α-Glucosidase

The α-amylase inhibitory activities of the extracted fractions were assessed based on a previous method with a few modifications [38]. A 25 µL sample (0.073–286.6 mg/mL) was mixed with 50 µL of α-amylase (0.1 U/mL) in a sodium phosphate buffer, after which 25 µL of starch solution (1%) was added into the mixture to start the reaction at 37 °C for 20 min. Then, the reaction was stopped by adding 125 µL of 3,5-dinitrosalicylic acid (DNS) reagent and incubating the mixture at 100 °C for 5 min. The absorbance of the reaction system was detected at 540 nm. Deionized water and acarbose were used as the control and reference standard, respectively. Acarbose was used as the positive control. Blank readings (no enzyme) were subtracted from each well, and the results were compared to the control. The inhibitory activity of α-amylase was calculated as follows: inhibition rate (%) = [(OD_control_ − OD_sample_)/OD_control_] × 100. The sample concentration providing 50% inhibitory activity (IC_50_) was calculated from the graph of the α-amylase inhibitory activity against the sample concentrations.

The α-glucosidase inhibitory activities of the extracted fractions were assessed based on a previous method, with a few modifications [39]. A total of 50 μL of α-glucosidase (0.2 U/mL), 550 μL of sodium phosphate buffer (pH 6.85), and 50 μL of sample (0.073–286.6 mg/mL) were mixed and reacted at 37 °C for 10 min. Subsequently, 50 μL of 4-N-nitrophenyl-α-d-glucopyranoside (PNPG, 5 mM) was added to the reaction system as a substrate. After incubation at 37 °C for 30 min, the reaction was stopped by adding 400 μL of Na_2_CO_3_ solution (0.2 M), and the absorbance was detected at 405 nm using a multi-mode microplate reader. Deionized water and acarbose were used as the control and reference standard. Blank readings (no enzyme) were subtracted from each well, and the results were compared to the control. The inhibitory activity of α-glucosidase was calculated as follows: inhibition rate (%) = [(OD_control_ − OD_sample_)/OD_control_] × 100. The sample concentration providing 50% inhibitory activity (IC_50_) was calculated from the graph of the α-glucosidase inhibitory activity against the sample concentrations.

#### 3.4.2. Cell Culture and Cell Viability Assay

HepG2 cells were cultured in Dulbecco’s modified Eagle’s medium (DMEM, phenol red-free) supplemented with 10% fetal bovine serum and 1% penicillin/streptomycin antibiotic, and incubated at 37 °C in a humidified incubator with 5% CO_2_.

The relative viability of the HepG2 cells was evaluated by the MTT method, as previously described [40]. Briefly, HepG2 cells were precultured in 96-well plates at 5 × 10^4^ cells per well for 24 h, and then treated with 0.5% ethanol (solvent control, SC), 2 mM metformin (positive control, Met), or one of the five extracted fractions for 24 h, separately. At the end of culturing, the culture medium was replaced with MTT–phosphate buffer solution (0.5 mg/mL) and incubated at 37 °C for 3 h; then, the MTT–phosphate buffer solution was discarded and 0.5 mL of dimethyl sulfoxide was added to the cells. The MTT value was determined by recording the absorbance of the extracts at 570 nm using a multi-mode microplate reader. The cell viability is presented as the percentage of the MTT value normalized to the MTT value of the blank control. The highest concentration of each extracted fraction that showed no significant difference compared with control group in cell viability was considered as the maximum nontoxic concentration. The subsequent assay of glucose consumption in cells was conducted based on the maximum nontoxic concentration for each extracted fraction.

#### 3.4.3. Induction of the Insulin-Resistance Model

HepG2 cells were precultured in 96-well plates at 5 × 10^4^ cells per well for 24 h. Afterwards, the HepG2 cells were treated with a high glucose + high fat solution containing 30 mM glucose and 0.2 mM palmitic acid to induce the IR-HepG2 model.

#### 3.4.4. Glucose Consumption Assay

HepG2 and IR-HepG2 cells were treated with 0.5% ethanol (solvent control, SC), 2 mM metformin (positive control, Met), or one of the five extracted fractions for 24 h, separately, and then the cells were treated with high glucose DMEM (30 mM glucose, serum-free, phenol red-free) for 12 h. The glucose consumption was determined by assaying the glucose concentration in the culture medium using a glucose determination kit with an absorbance at 505 nm using a multi-mode microplate reader. Glucose consumption = 30 mM–cell supernatant glucose content.

### 3.5. Compound Analysis of the Extraction Phase

A total of 100 mg of extract was dissolved in 1 mL of 70% methanol and filtered with a 0.22 µm filter membrane. Then, a 2 µL sample was injected into the LC–MS system. The sample was separated with HPLC (Ultimate 3000LC, Thermo Fisher Scientific, Waltham, MA, USA) equipped with a C18 chromatographic column (1.8 μm × 2.1 × 100 mm, Zorbax Eclipse C18, Agilent technologies, Santa Clara, CA, USA) by elution in gradient mode. The gradient elution method, employing the mobile phase A (water + 0.1% formic acid) and mobile phase B (acetonitrile), was as follows: 95% of eluent A at 0 min, 70% at 2 min, 22% at 7 min, 5% at 14 min, and 95% at 20 min, with a flow rate of 0.3 mL/min and column temperature of 30 °C.

Mass spectrometric analysis was performed with a Q Exactive HF mass spectrometer (Q Exactive HF, Thermo Fisher Scientific, Waltham, MA, USA). The electrospray spray ion source (ESI) conditions were as follows: negative ion scanning mode, and vaporizer and capillary temperatures of 325 and 330 °C, respectively. The spray voltage was 3.5 kV. Full mass spectra were obtained in the 100–1500 *m*/*z* range at a resolution of 120,000. The data-dependent dd-MS^2^ spectra were acquired at a resolution of 60,000, and the top 10 precursors were selected for fragmentation by high-energy collusion dissociation (HCD). The total ion current (TIC) chromatogram was recorded.

The Compound Discoverer 3.2 software was used for retention time correction, peak identification, and peak extraction. The Thermo mzCloud online database and Thermo mzValut local database were used for substance identification according to secondary mass spectrometry information. Identified compounds were classified into 28 groups. The relative content of each classification was calculated by relative peak area (total peak area of each group divided by the total area of all peaks).

### 3.6. Statistical Analysis

Three to six independent experiments were carried out for each analysis. The data are expressed as the means ± SD. The statistical significance and differences were determined with one-way analysis of variance (ANOVA), followed by Dunnett’s *t*-test.

## 4. Conclusions

The different extracted fractions from Pingguoli pear fermentation broth displayed antioxidant and hypoglycemic activities in a dose-dependent manner. The EAF showed the highest antioxidant activity compared to other four extracted fractions, and was able to significantly inhibit α-amylase and α-glucosidase activities, as well as significantly improve the glucose consumption of HepG2 cells. The composition analysis showed that the EFA contained 3′-C-glucosylisoliquiritigenin, robustside D, caffeic acid, and chlorogenic acid, which are potential candidates for its antioxidant and hypoglycemic activities. Therefore, further separation, purification, and material structure identification of the EAF should be carried out in the future, and its mechanism of action, as well as the synergy among different bioactive compounds, should be further studied to develop functional edible products with antidiabetic effects.

## Figures and Tables

**Table 1:** Content of extracts from Pingguoli pear fermentation extracted by different solvents.

Extracted Fraction	PEF	CF	EAF	NF	WF
Extract content (mg/mL Pingguoli pear fermentation broth)	5.36 ± 1.28	6.42 ± 1.08	1.36 ± 0.23	23.58 ± 2.79	573.23 ± 41.16

Values are mean ± SD (*n* = 3).

**Table 2:** Antioxidant activity of the five extracted fractions from Pingguoli pear fermentation broth.

Fractions	IC_50_ (mg/mL)		
	DPPH	ABTS	FRAP
PEF	16.642 ± 2.342 ^b^	27.583 ± 4.312 ^b^	7.176 ± 1.014 ^a^
CF	3.205 ± 0.471 ^c^	3.160 ± 0.495 ^c^	1.824 ± 0.042 ^b^
EAF	0.238 ± 0.052 ^d^	0.293 ± 0.032 ^d^	0.193 ± 0.022 ^c^
NF	4.100 ± 0.639 ^c^	2.421 ± 0.458 ^c^	1.706 ± 0.095 ^b^
WF	55.076 ± 3.753 ^a^	252.122 ± 28.601 ^a^	8.235 ± 0.743 ^a^
Ascorbic acid	0.004 ± 0.001 ^e^	0.064 ± 0.017 ^e^	0.073 ± 0.006 ^d^

Values are mean ±SD (*n* = 3). Values with different letters represent statistically different at *p* < 0.05.

**Table 3:** IC_50_ values of α-amylase and α-glucosidase inhibition by five extracted fractions from Pingguoli pear fermentation broth.

Parameter	Fraction					Acarbose
	PEF	CF	EAF	NF	WF	
α-Amylase inhibition (IC_50_, mg/mL)	2.95 ± 0.33 ^d^	8.04 ± 0.75 ^c^	0.34 ± 0.04 ^e^	25.41 ± 3.94 ^b^	174.57 ± 11.32 ^a^	0.19 ± 0.01 ^f^
α-Glucosidase inhibition (IC_50_, mg/mL)	7.89 ± 0.34 ^b^	3.15 ± 0.31 ^c^	0.95 ± 0.19 ^e^	3.62 ± 0.27 ^c^	248.58 ± 19.63 ^a^	1.35 ± 0.07 ^d^

Values are mean ± SD (*n* = 3). Values with different letters represent statistically different at *p* < 0.05.

**Table 4:** Relative cell viability of different extracted fractions at their maximum nontoxic concentration.

Sample	BC *	SC ^#^	Met	IR Model ^&^	PEF	CF	EAF	NF	WF
Maximum nontoxic concentration (µg/mL)	—	—	258.3	—	133.0	2.5	18.0	628.0	28660
Relative cell viability (%)	100.0 ± 4.3	98.2 ± 2.4	97.3 ± 3.8	99.0 ± 5.4	101.4 ± 3.5	98.5 ± 4.7	99.2 ± 2.3	99.7 ± 5.4	98.1 ± 1.4

* BC represents blank control, cells treated with medium; **^#^**: SC represents solvent control, cells treated with medium contain 0.5% ethanol; **^&^**: IR model, cell treated with 30 mM glucose and 0.2 mM palmitic acid. Values are mean ± SD (*n* = 6).

**Table 5:** Identification of ten main compounds in the EAF from Pingguoli pear fermentation broth by HPLC-MS/MS.

No.	Compound Name	Formula	Category	Mw	RT (min)	Relative Concentration (μg/mL)
1	d-(+)-Galactose	C_6_H_12_O_6_	Organooxygen compounds	180.1	0.781	858.0
2	3’-C-Glucosylisoliquiritigenin	C_21_H_22_O_9_	Linear 1,3-diarylpropanoids	418.1	7.208	591.9
3	Robustaside D	C_21_H_22_O_10_	Saccharolipids	434.1	6.662	244.2
4	Caffeic acid	C_9_H_8_O_4_	Cinnamic acids and derivatives	180.0	6.001	227.3
5	Ethyl α-d-glucopyranoside	C_8_H_16_O_6_	Organooxygen compounds	208.1	1.316	201.9
6	α-Methyl d-mannoside	C_7_H_14_O_6_	Organooxygen compounds	194.1	0.843	187.3
7	Chlorogenic acid	C_16_H_18_O_9_	Organooxygen compounds	354.1	5.692	182.2
8	Dimethyl citrate	C_8_H_12_O_7_	Carboxylic acids and derivatives	220.1	4.556	180.1
9	l-Iditol	C_6_H_14_O_6_	Organooxygen compounds	182.1	0.763	129.0
10	α,α-Trehalose	C_12_ H_22_ O_11_	Organooxygen compounds	342.1	0.796	112.9

## Data Availability

The data presented in this study are available on request from the corresponding author.
